# Interaction Potency of Single-Walled Carbon Nanotubes with DNAs: A Novel Assay for Assessment of Hazard Risk

**DOI:** 10.1371/journal.pone.0167796

**Published:** 2016-12-09

**Authors:** Chunhe Yao, Cristina Carlisi, Yuning Li, Da Chen, Jianfu Ding, Yong-Lai Feng

**Affiliations:** 1 Exposure and Biomonitoring Division, Environmental Health Science and Research Bureau, Environmental and Radiation Health Sciences Directorate, Healthy Environments and Consumer Safety Branch, Health Canada, Ottawa, Ontario, Canada; 2 Department of Chemical Engineering, Department of Chemistry, and Waterloo Institute for Nanotechnology (WIN), University of Waterloo, Waterloo, Ontario, Canada; 3 Cooperative Wildlife Research Laboratory and Department of Zoology, Southern Illinois University, Carbondale, Illinois, United States; 4 Security and Disruptive Technologies, National Research Council of Canada, Ottawa, Ontario, Canada; Queen's University at Kingston, CANADA

## Abstract

Increasing use of single-walled carbon nanotubes (SWCNTs) necessitates a novel method for hazard risk assessment. In this work, we investigated the interaction of several types of commercial SWCNTs with single-stranded (ss) and double-stranded (ds) DNA oligonucleotides (20-mer and 20 bp). Based on the results achieved, we proposed a novel assay that employed the DNA interaction potency to assess the hazard risk of SWCNTs. It was found that SWCNTs in different sizes or different batches of the same product number of SWCNTs showed dramatically different potency of interaction with DNAs. In addition, the same SWCNTs also exerted strikingly different interaction potency with ss- versus ds- DNAs. The interaction rates of SWCNTs with DNAs were investigated, which could be utilized as the indicator of potential hazard for acute exposure. Compared to solid SWCNTs, the SWCNTs dispersed in liquid medium (2% sodium cholate solution) exhibited dramatically different interaction potency with DNAs. This indicates that the exposure medium may greatly influence the subsequent toxicity and hazard risk produced by SWCNTs. Based on the findings of dose-dependences and time-dependences from the interactions between SWCNTs and DNAs, a new chemistry based assay for hazard risk assessment of nanomaterials including SWCNTs has been presented.

## Introduction

Single-walled carbon nanotubes (SWCNTs) are one-dimensional nanomaterials, which take the form of a single graphene sheet rolled into a tube with nanometer-sized diameters [1,2]. They possess remarkable thermal properties, photo-stability, large surface area to volume ratio, tensile strength, and electrical properties. Owing to these unique properties, they have been used in a variety of consumer and industrial products, such as electronics, protective clothing, drug delivery systems, and electrochemical sensors [3–7]. Along with a rapidly growing use of SWCNTs, their migration into the environment during the processes of production, delivery, use, and disposal has raised concerns on their potential hazards to humans and the environment [8,9]. In a previous study, considerable amounts of SWCNTs were detected in atmosphere and on gloves in occupational settings, indicating potential inhalation and dermal exposure of these nanomaterials [10]. Therefore, the hazards of SWCNTs to human health and environmental quality require careful investigations.

Traditional toxicity assays, including the comet assays, are the common approaches to assessing hazard risks produced by nanomaterials, such as SWCNTs. The toxicity of SWCNTs and other nanomaterials to humans is believed to originate from the physicochemical interaction with biomolecules [11–13]. Therefore, the elucidation of the physical characteristics (e.g. size and mass distribution) and chemical compositions of a nanomaterial is the key element for hazard risk assessment of nanomaterials. However, nanomaterials including SWCNTs are usually mixtures of entities with large variation in geometry, dimensions, isomers, chemical impurities, and structural defects [14]. Moreover, the nanomaterials with identical shape and size, but produced by different processes or from different batches, may exhibit dramatically different properties, such as electrical conductivity, charge mobility, and toxicity [15–18]. Many previous studies showed that common physicochemical parameters of SWCNTs, such as size, mass, shape, surface area, zeta potential and chemical composition, did not exhibit convincing correlations with toxicity, likely due to the structural complexities [19–23]. This consequently raises a challenge to the hazard risk assessment of nanomaterials. Furthermore, the traditional assays are greatly confined since the necessary information of most nanomaterials couldn’t be quickly provided for assessment of their hazard risk. Considering the wide diversity of nanomaterials and observations that various nano-forms with the same chemical composition possess diverse toxicological properties, new approaches that do not rely on conventional toxicity testing methods should be developed to better assess the hazards of nanomaterials including SWCNTs.

SWCNTs were reported to result in reactive oxygen species that could damage DNA, proteins, and membranes in cells [24,25]. The DNA damage can be in the form of single- and double-strand breaks, loss of excision repair, cross-linking, alkali-labile sites, point mutations, and structural and numerical chromosomal aberrations by interactions of chemicals and substances with DNA, which are all important information to genotoxicity. The comet assay was commonly utilized to assess the genotoxic risk of a chemical or substance [26]. Our group recently used DNA probes to explore interactions between DNA and various environmental pollutants as a means to assess chemicals based on their interaction potency with DNA [27,28]. In this work, we reported a new approach assessing the genotoxic effect of SWCNTs by addressing the interaction potency and rates of SWCNTs with DNA oligonucleotides, and this new developed assay could be also applied to assessment of various other nanomaterial and nanocomposites.

## Materials and Methods

### Chemicals

Tris(hydroxymethyl)aminomethane (Tris, 99.9+%), hydrochloric acid (HCl, 36.5–38.0%), triethylamine (TEA, 99.5+%), triethylammonium acetate solution (TEAA, 1.0 M, pH 7.0) and acetonitrile (ACN, 99.9+%) were purchased from Sigma-Aldrich Canada (Oakville, ON, Canada). Unmodified oligonucleotides with sequences either complementary to the human serotonin transporter gene 5-TGGACCTGGGCAATGTCTGG-3 (5-HTT) or its complement, 5’-CCAGACATTGCCCAGGTCCA-3’ (RC-5-HTT) were purchased from Sigma-Genosys Canada (RP1 purity, Oakville, ON, Canada). Tetrahydrofuran (THF, 99.9+%, HPLC grade, inhibitor-free) was purchased from Sigma-Aldrich (St. Louis, MO, USA). Deionized water (DIW, 18.3 MΩ cm) was made in-house using a Milli-Q Integral Water Purification System from EMD Millipore (Billerica, MA, USA).

All single-walled carbon nanotubes (SWCNTs) were purchased from Sigma-Aldrich. They had a CAS number of 308068-56-6 but different product numbers: 704148 batch A, 704148 batch B, 704113, and 773735, which were denoted as SWCNT1A, SWCNT1B, SWCNT2, and SWCNT3, respectively. Table 1 shows some of their property parameters. All SWCNTs were used as received.

**Table 1:** Properties of SWCNTs.

Carbon nanotubes	Cat. No.	Batch	Diameter (nm)	Length (nm)	Content (carbon as SWCNT)
SWCNT1A	704148	Lot# MKBN4897V Pcode 1001512184	0.7–0.9	900	≥77%
SWCNT1B	704148	Lot# MKBJ6336V Pcode 1001237310	0.7–0.9	900	≥77%
SWCNT2	704113	Lot# MKBK1401V Pcode 1001311603	0.7–1.3	800	≥70%
SWCNT3	773735	Lot# MKBN5945V Pcode 1001515737	0.7–0.9	1500	≥93%

### Preparation of solutions

Stock solutions of 0.8 mM 5-HTT and 0.8 mM RC-5-HTT were prepared in deionized (DI) water, transferred into 1.5-mL Eppendorf tubes and stored at −80°C before use. The working solution of 0.4 mM RC-5-HTT was prepared by diluting the above stock solution with DI water and stored at −20°C. The synthesis of 0.4 mM double-stranded DNA (ds-DNA) was followed by the previous method [24]. Briefly, equal volumes of the above stock 5-HTT and RC-5-HTT solutions (0.8 mM) were added into a 1.5-mL Eppendorf tube. The tube was vortexed, heated in a boiling water bath (1000-mL beaker) for 5 min, and then allowed gradually cooling down to room temperature (~4 h). The annealed ds-DNA was stored at −20°C before use. A stock solution of 5% TEA (v/v) in THF was prepared and stored at room temperature. A stock solution of 1 M Tris-HCl buffer was prepared in DI water (pH = 7). All solvents used for HPLC analysis were of HPLC grade or better.

The SWCNT1B dispersion solution was prepared by adding 100 mg of SWCNT1B into 20 mL of 2% sodium cholate solution. The solution was then homogenized by tip sonication with a Sonifier (Branson Sonifier 250, maximum power, 200W) for 30 min or 8 h at 30°C with a diameter of 10 mm tip that was operated at a duty cycle of 40% and power output of 40% to give an SWCNT1B dispersion solution of 5 mg mL^-1^

### Interaction of DNAs with SWCNTs

To examine the interactions between DNAs and SWCNTs, 862.5 μL of DI water, 50 μL of 1 M Tris-HCl buffer (pH = 7), 50 μL of THF solution containing 5% (v/v) triethylamine, and 37.5 μL of 0.4 mM of either ss-DNA (RC-5-HTT) or ds-DNA solution were mixed in a 1.5-mL Eppendorf tube. The mixture was gently vortexed, followed by the addition of 0, 0.5, 1.0, 2.0, 4.0, or 5.0 mg of SWCNTs. The mixture was immediately vortexed again and then incubated in a Thermomixer-R auto-shaker (Eppendorf, Westbury, NY, USA) at 37°C with 600 rpm in the dark for 48 h. The interaction was terminated by centrifugation at 7000 rpm for 10 min using a microcentrifuge (Model 5424, Eppendorf, Westbury, NY, USA). The supernatant was subjected to HPLC analysis. All interaction experiments were repeated for three times.

For the study of interaction rates, the solution was prepared in the same manner as above before the addition of SWCNTs. The SWCNTs (1 mg for ss-DNA and 4 mg for ds-DNA) were added, and the mixtures were incubated for 2, 5, 10, 15, 20, 30, 40, 60, 120, 240, or 360 min, respectively. The interaction was terminated by the aforementioned centrifugation procedure, and the supernatant was subjected to HPLC analysis. A zero minute interaction time was made using a mixture prepared in the same manner as above but without the addition of the test SWCNTs. The blank control included all buffers and solvents but without the addition of DNA and SWCNTs. All experiments were repeated for three times.

To examine interactions between the dispersed SWCNT1B and DNAs, a mixture of 62.5 μL of DI water, 50 μL of 1 M Tris-HCl buffer at pH 7.0, 50 μL of THF solution containing 5% (v/v) triethylamine, and 37.5 μL of 0.4 mM ss-DNA (RC-5-HTT) or ds-DNA was prepared in a 1.5-mL Eppendorf tube. Then 0.8 mL of SWCNT1B dispersion solution with a concentration varying from zero to 5 mg mL^-1^ in 2% sodium cholate (specifically, 0, 0.625, 1.25, 2.5 and 5 mg mL^-1^) was added into the tube. The mixtures were incubated for 48 h with the same conditions as mentioned above. The incubation was terminated using the same centrifugation procedure described above, and then a portion of the upper solution was subjected to HPLC analysis. The rate of the interaction between the dispersed SWCNT1B and DNAs was investigated by adding the SWCNT1B dispersion solution (5 mg mL^-1^) to make a final concentration of 1 mg mL^-1^ of SWCNT1B in the reaction vial. The interaction times were 3, 5, 10, 15, 25, 45, 70, or 135 min, respectively.

### Measurement of DNAs and interaction products

An Agilent 1200 HPLC System (Agilent Technologies) with a Supelcosil LC-18-DB column (3 μm, 250 mm × 2.1 mm) and a Supelguard TM LC-18-DB guard column (3 μm, 20 mm × 2.1 mm) was used for the separation of DNAs and interaction products, followed by the detection using a diode-array detector (DAD) at a wavelength of 258 nm. The column temperature was 30°C. The injection volume of sample was 10 μL. The mobile phases of A (100 mM TEAA in water at pH 7.0) and B (100 mM TEAA in 50% (v/v) ACN at pH 7.0) were set in a linear gradient beginning with 15% B to 30% B in 20 min. This was followed by an increase to 60% B in 10 min and then to 100% B in 5 min. After an isocratic elution at 100% B for 13 min, the mobile phase was shifted back to 15% B in 2 min. The mobile phases had a flow-rate of 0.22 mL min^-1^.

## Results and Discussion

### Interaction of SWCNTs with DNAs

**Fig 1:**
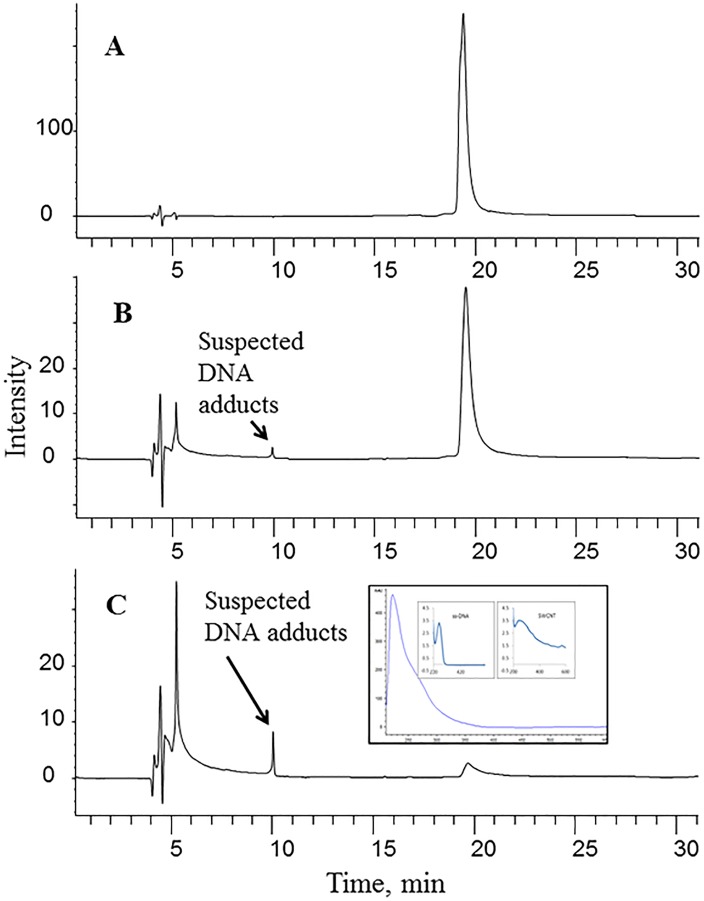
Typical HPLC chromatogram of the interaction of SWCNTs with the ss-DNA oligonucleotides. Carbon nanotubes: SWCNT3 (0.7–0.9 nm in diameter, 1500 in length, purity≥95%, DNA: ss-DNA oligonucleotides (RC-5-HTT), 0.015 μmole. Total volume for interaction solution: 1 mL. (A) no SWCNT3, (B) 2 mg SWCNT3, (C) 5 mg SWCNT3. Main insert: the UV spectra of suspected interaction products. Small right insert: the UV spectra of ss-DNA. Small left insert: the UV spectrum of SWCNT3 dispersed in 2% of sodium cholate solution.

An interaction between a nanomaterial and DNA oligonucleotides can occur via chemical or physical means [29–35]. In the present study, the results showed that the intensity of the ss-DNA peak at 19.5 min decreased when the amount of SWCNTs was increased (Fig 1; SWCNT3 as an example). Meanwhile, a new peak appeared at a retention time of 9 min concurrently with the decrease in the intact DNA peak intensity. This new peak intensified with increasing concentration of SWCNTs (Fig 1B and 1C), suggesting that it may represent a new product formed from the interaction between SWCNTs and DNA. The UV spectrum of the new product resembled the sum of the absorption profiles of DNA and SWCNTs (Fig 1C and 1C inserts), which is very similar to the UV spectra of the DNA-SWCNT adducts reported previously [36]. Therefore, it is reasonable to assume that this new peak is a DNA-SWCNT interaction product.

One possible driving force for the interaction between SWCNTs and DNA is the interaction of π electrons on the surface of SWCNTs with those in the building blocks of DNA (cytosine (C), guanine (G), adenine (A), or thymine (T)) [37,38]. The rapid decrease of the intact DNA peak indicated that the interaction between SWCNTs and DNA molecules was very strong. We noticed that the area of the newly formed peak was much smaller, compared to the reduction in the intact DNA peak area. This discrepancy might be due to the poor solubility of the DNA-SWCNT product. Thus, only a small soluble fraction of this product was present in the supernatant subject to HPLC analysis.

SWCNTs are hydrophobic and insoluble in water. Therefore, the DNA-SWCNT interaction product was expected to be more hydrophobic than the DNA oligonucleotides in the reaction mixture, and the interaction product was supposed to have a longer retention time than the DNA oligonucleotides on the C18 HPLC column in reversed-phase chromatography. However, this new peak showed at a shorter retention time than the intact DNA oligonucleotides, suggesting that this adduct was more hydrophilic than the intact DNA oligonucleotides. We speculate that the relatively more polar product might be produced via the interaction of the π electron-containing bases in DNA oligonucleotides with SWCNTs through the π-π interaction as described previously [39], which forced the more hydrophilic phosphate groups to be on the surface of the DNA-SWCNT adduct rendering the DNA-SWCNT interaction product (DNA-wrapped SWCNTs) more hydrophilic.

### DNA interaction potency

**Fig 2:**
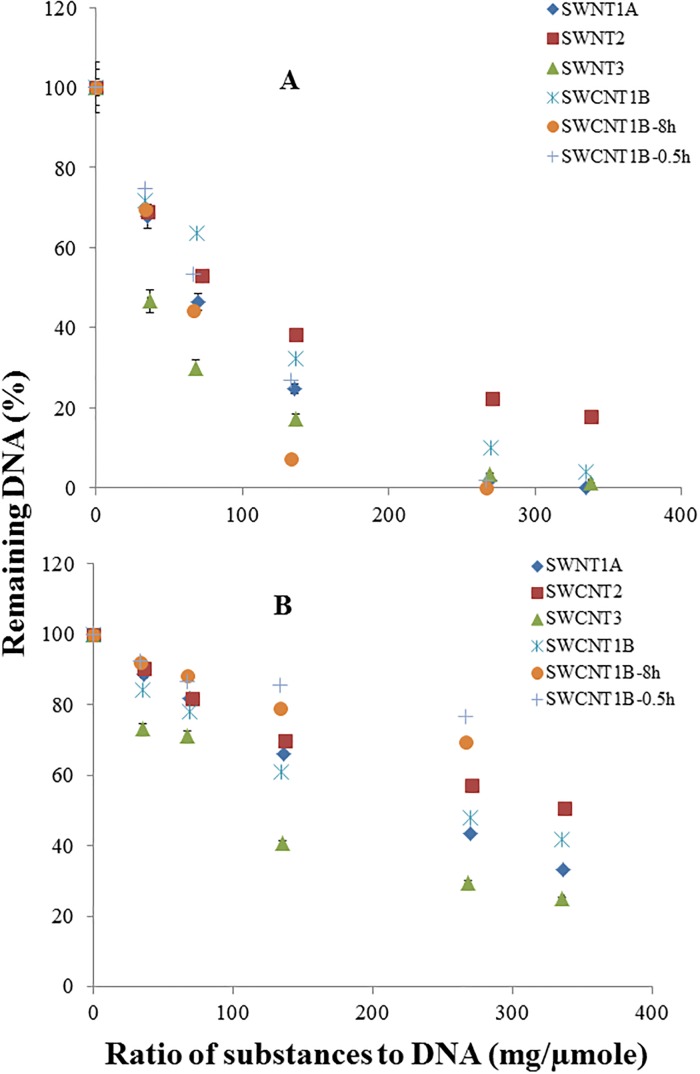
Dose-response curves for interaction of SWCNTs with DNAs. (A) DNA: ss-DNA (RC-5-HTT), 0.015 μmole. Total volume for interaction solution: 1 mL. (B) DNA: ds-DNA (ds-5-HTT), 0.015 μmole. Total volume for interaction solution: 1 mL.

Fig 2 depicts concentration changes of the intact DNA oligonucleotides with increasing amount of SWCNTs. Overall, the concentration of the intact DNA oligonucleotides remained in the reaction mixture decreased with increasing amount of SWCNTs for all four types of the test SWCNTs (SWCNT1A, SWCNT1B, SWCNT2, and SWCNT3). Specifically, SWCNT3 showed the largest reduction in the intact DNA concentration at any given SWCNT/DNA ratio ranging from 1 to 320, while SWCNT 2 was the least effective. Curves were fitted to the SWCNT and DNA interaction data in Fig 2 using the SigmaPlot^®^ four-parameter fit equation which was utilized to evaluate the reactivity of a chemical with DNAs in our previous reports [27,28]. From these equations, we calculated the amount of SWCNTs required inducing a 50% reduction in the original amount of DNA oligonucleotides in the reaction mixture, referred to as the Effective Dose 50 (ED50) with a unit of mg/μmol (SWCNTs/DNAs) (Table 2). The ED50 values can represent the potency of SWCNTs interacting with DNAs, *i*.*e*. the smaller value of ED50, the stronger interaction potency. The ED50 values of the test SWCNTs were found to be in the order of SWCNT3 < SWCNT1A < SWCNT1B < SWCNT2, regardless of the type of DNA used (ss-DNA or ds-DNA). Surface chemistry and surface reactivity at the surface of carbon nanotubes was reported to play an important role in the cytotoxic effect of carbon nanotubes [39]. SWCNT3 has the smallest ED50 value compared to other SWCNTs, likely suggesting the strongest surface reactivity or the strongest surface chemistry. SWCNT3 has the highest purity (>93%), narrowest diameter distribution and the longest length that gives the highest aspect ratio (length to diameter). Thus, the highest interaction potency or risk is expected among the three SWCNTs. In contrast, SWCNT2 has the widest diameter distribution, the lowest purity and the shortest length that gives the lowest aspect ratio, and therefore, the lowest interaction potency or risk is expected. This result is complying with the previous reported results on effects of SWCNTs in Swiss rice, which was profiling as a function of dose, length and surface chemistry [39]. Interestingly, SWCNT1A and SWCNT1B, which were manufactured in different batches from the same vendor, had the same purity and other physicochemical parameters according to the supplier (Table 1), but they showed very different ED50 values. These results indicated that common physicochemical parameters of SWNCTs (probably also true for other nanomaterials) are insufficient to determine their interaction potency (strength) with DNA. Although some dose-dependent results were reported previously for evaluation of cytotoxicity of SWCNTs [39], the toxicity difference between different batches of the same SWCNTs has not been reported yet. In this study, the ED50 values can differentiate the interaction potency of all test SWCNTs with the DNA oligonucleotides, including between different batches. Therefore, this chemistry based assay with ED50 values could be an alternative tool to other toxicity assays in assessing the hazard risk of SWCNTs to human and the environment.

**Table 2:** Interaction potency data of SWCNTs with DNAs.

DNA type	Parameter^a^	Non-dispersed SWCNTs				Dispersed SWCNTs	
		SWCNT1A	SWCNT1B	SWCNT2	SWCNT3	SWCNT1B-0.5h^c^	SWCNT1B-8h^d^
**ss-DNA**	Min.	-24.8	-55.2	-14.0	-18.6	-33.0	-8.8
	Max.	100.0	99.5	100.0	100.0	100.0	99.4
	EC50	90.9	196.3	111.4	45.9	112.3	60.2
	Hillslope	-1.123	-0.956	-0.857	-0.813	-1.194	-1.824
	R^2^	0.9992	0.9921	1	0.9994	1	0.9936
	ED50 (mg/μmole)^b^	59	81	84	31	72	51
**ds-DNA**	Min.	-210.4	4.9	18.1	1.4	72.3	30.8
	Max.	99.9	99.9	100.1	99.4	100.0	99.9
	EC50	1429.09	213.88	234.9	112.7	71.4	344.0
	Hillslope	-0.899	-0.9537	-1.045	-1.087	-1.285	-0.916
	R^2^	0.9996	0.995	0.9987	0.9766	1	0.9979
	ED50 (mg/μmole)^b^	228	248	347	126	1171	627

^a^Parameters determined by four parameter logistic fit in SigmaPlot, *y = min*. *+ (max*.*—min*.*)/(1 + (x/EC50)*^*-Hillslope*^*)*, where min = min(y), max = max(y), *EC50 = x50(x*,*y)* and *Hillslope = sign(x*,*y)*.

^b^ED50 = the effective dose of SWCNTs required inducing a 50% reduction of the text DNA (unit: mg/μmole). Every point on the curves in the figures is the average of triplicate results with a RSD ≤ 5%.

^c^SWCNT1B-0.5h dispersion, 0.5 h sonication time.

^d^SWCNT1B-8h dispersion, 8 h sonication time

Fig 2 also showed that all types of SWCNTs had significantly higher interaction potency with ss-DNA than with ds-DNA. Specifically, the ED50 values of SWCNTS to ds-DNA were much greater compared to those of SWCNTs to ss-DNA as shown in Table 2. SWCNTs were suggested to interact with DNA molecules through π-π interaction (or π-π stacking) with the DNA molecules wrapped around SWCNTs [29–35]. When DNA molecules interact with SWCNTs, the aromatic nucleotide bases need to orient with their planes parallel to the surface of the SWCNTs to form π-π stacking [37,38]. Compared to ss-DNA, the aromatic nucleotide bases in ds-DNA are more difficult to orient parallel to the surface of the nanotube due to the strong hydrogen bonds between the complementary bases in ds-DNA. Consequently, the interaction of ds-DNA with SWCNTs is hindered compared to ss-DNA, resulting in a weaker interaction potency of the former. Although the interaction was not characterized with imaging techniques such as ATOM and SEM in this study to support the π-π interaction mechanism proposed, this π-π interaction mechanism can well explain different interaction potency of SWCNTs with ss-DNA and ds-DNA observed in this study, which could be considered as a self-evidence of this proposal.

### Interaction potency of SWCNT dispersions with DNAs

The exposure medium of SWCNTs is important to assessing the toxicity of SWCNTs since the SWCNTs have a strong tendency to agglomerate following intratracheal exposures [39]. When SWCNTs are dispersed in a liquid medium, they were hypothesized to interact with DNA molecules more effectively due to the increased chance for SWCNTs to encounter DNA molecules. A longer sonication time would result in a more homogenous dispersion, leading to their stronger interaction with DNAs. SWCNT1B was chosen as an example to be dispersed in 2% sodium cholate solution for 0.5 h and 8 h to make two dispersions, SWCNT1B-0.5h and SWCNT1B-8h, respectively. As expected, the ss-DNA concentration dropped more rapidly for both dispersed SWCNT1B samples than the non-dispersed SWCNT1B (Fig 2A). Additionally, the ss-DNA concentration decreased faster when reacting with SWCNT1B-8h than SWCNT1B-0.5h. The ED50 values were 81, 72 and 51 mg/μmol for the non-dispersed SWCNT1B, SWCNT1B-0.5h and SWCNT1B-8h, respectively, demonstrating a dispersion effect on the interaction potency (strength) of SWCNT1B with ss-DNA (Table 2). Surprisingly, a much slower drop in the DNA concentration was observed when the SWCNT1B-0.5h dispersion was added to a ds-DNA solution (Fig 2B), which resulted in a much greater ED50 value (1171 mg/μmol) than that of the non-dispersed SWCNT1B (248 mg/μmol) (Table 2). Although a longer sonication time for the preparation of SWCNT1B dispersion appeared to decrease the ED50 value, the value 627 mg/μmol for the interaction of SWCNT1B-8h with the ds-DNA is more than double of the ED50 value for the interaction between its solid phase and ds-DNA, which is greatly different with the case of the interaction with ss-DNA. The differential effects of dispersion of SWCNT on the reaction with ss- and ds-DNAs could be explained as follows. SWCNTs in the dispersion are wrapped by the stabilizer sodium cholate, which would hinder the interaction of SWCNTs with the DNA molecules and offset the effect of the increased exposure of dispersed SWCNTs to DNAs. However, it was reported that when ss-DNA oligomers were added to sodium cholate-dispersed SWCNTs, exchange reactions between ss-DNA and sodium cholate occurred [40]. The equilibrium constants of the 20-mer ss-DNA that is with the same length of the ss-DNA used in this study were found to be in a range of 1260 to 8160, indicating that ss-DNA could readily replace sodium cholate molecules. Therefore, the interaction reactivity between SWCNTs and ss-DNA was negligibly affected, and the overall effect of dispersion of SWCNTs on their interaction potency (strength) with ss-DNA was increased due to the increased exposure of SWCNTs to ss-DNA molecules. The longer sonication time also resulted in increased interaction strength. On the other hand, the interaction potency (strength) of SWCNTs with ds-DNA was much weaker as discussed above. Therefore, the equilibrium constants of the exchange reaction between ds-DNA and sodium cholate are expected to be smaller, making the replacement of the sodium cholate molecules adsorbed on SWCNTs with ds-DNA molecules more difficult. As a result, SWCNT1B-0.5h showed a much greater ED50 (or much weaker interaction strength) with ds-DNA even though the dispersion increased the chance of (sodium cholate-wrapped) SWCNTs to encounter ds-DNA molecules. With a longer sonication time (8 hours), the equilibrium constants of exchange reactions would remain the same. However, the improved dispersion of SWCNTs would increase their interaction likelihood with ds-DNA, leading to a reduction in ED50 to 627 mg/μmol. Hence, above observations demonstrated that the interaction potency of SWCNTs with DNAs varied with different exposure media, and this difference suggested a different hazard risk of SWCNTs when they were exposed in different media.

### Interaction rates of SWCNTs with DNAs

In chemistry, interaction rate defined as how quickly or slowly a reaction takes place. The interaction rate of SWCNTs with DNAs may reflect the toxicity or the hazard risk of SWCNTs for acute exposure.

**Fig 3:**
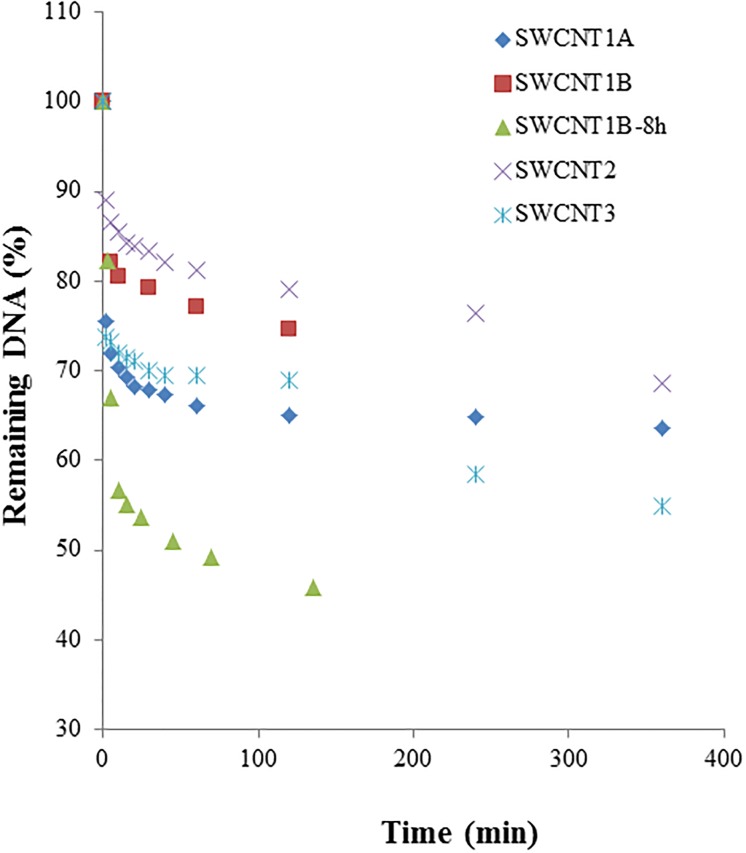
Time-dependent relationship of SWCNTs to DNAs. DNA: ss-DNA (RC-5-HTT), 0.015 μmole. Total volume for interaction solution: 1 mL. SWCNTs, 1 mg.

Fig 3 shows the changes of the ss-DNA concentration over time in the presence of different SWCNTs. The results suggested that all SWCNTs showed different interaction rates. Specifically, the interaction rate of SWCNT1A was the fastest, and the interaction rate of SWCNT2 was the slowest. Similar to the influence of dispersion on the interaction potency, when the SWCNT1B was dispersed in a 2% sodium cholate solution, the interaction rate was significantly increased (Fig 3A), which might indicate potential increase of toxicity or hazard risk for the nanomaterials when they are exposed in liquid media. The interaction rate order of different SWCNTs with ss-DNA was SWCNT1B-8h > SWCNT1A > SWCNT3 > SWCNT1B > SWCNT2 based on their curve slopes, which is in a different order with their DNA interaction potency. This difference might be explained by the difference between acute risk and chronic risk of SWCNTs on DNA damage. Specifically, the interaction rate of SWCNT1B-8h was the fastest, likely indicating a risk of fast DNA damage tendency compared to SWCNT3 and SWCNT1A whose ED50 values were larger than SWCNT1B-8h. These results might suggest that the dose-dependent results were not enough for evaluation of hazard risk of nanomaterials.

The differences in interaction rates observed for the non-dispersed and dispersed SWCNT1B are likely due to the fact of the increased exposure of SWCNTs to ss-DNA molecules as discussed above for non-dispersed SWCNT1B. The rates of interaction of different SWCNTs with ds-DNA were in the same order as those of SWCNTs with ss-DNA. Toxicity is a measure of the poisoning strength of a chemical or substance, which is the result of reaction rate and the amount of substances or chemicals with biomolecules in life. In general, the fast interaction rate of SWCNTs with DNAs indicates that SWCNTs could alter the natures of DNAs fast, which gives high potential risk to the DNAs. Therefore, the interaction rate between SWCNTs with DNA could be used as the indicator of toxicity for acute exposure. Considering the strong need for establishing an international set of guidelines and protocols for the determination of the cytotoxicity of SWCNTs *in vitro* and *in vivo* [39], the ED50 values for interaction potency and the interaction rate could be potential useful evaluating criteria for nanotoxicity.

## Conclusions

In the present study, we have developed a novel chemistry based assay to assess the hazard risk of SWCNTs based on their potency and rate of interaction with DNA. The new approach can distinguish interaction strength (potency) between different SWCNTs as well as the same SWCNTs between different batches. The dispersion of the SWCNTs in liquid medium significantly enhanced the interaction strength (potency) with DNAs, implying that the exposure media may significantly affect the hazard risk of SWCNTs to humans. To the best of our knowledge, this is the first chemistry based assay with the concept of DNA interaction potency and rate for hazard risk assessment.
